# Dataset for techno-economic analysis of catalytic hydrothermolysis pathway for jet fuel production

**DOI:** 10.1016/j.dib.2021.107514

**Published:** 2021-10-27

**Authors:** Sudha Eswaran, Senthil Subramaniam, Scott Geleynse, Kristin Brandt, Michael Wolcott, Xiao Zhang

**Affiliations:** a Bioproducts Sciences and Engineering Laboratory, Washington State University, 2710 Crimson Way, Richland WA 99354 United States; b Voiland School of Chemical Engineering and Bioengineering, Washington State University United States; c Composite Materials Engineering Centre, Washington State University, P.O. Box 645815, Pullman WA 99164 United States

**Keywords:** Biofuel, Techno-economic analysis, Catalytic hydrothermolysis, Conversion technologies, Sustainable aviation fuel

## Abstract

This detail the economics of Catalytic Hydrothermolysis (CH), an approve pathway for sustainable aviation fuel (SAF) production. Techno-economic analysis was conducted with the assumption of CH processing facility that process 832 metric tonnes per day of feedstock into renewable fuels such as SAF, gasoline and diesel. Economic data includes estimation of renewable fuel production plant cost such as capital and operating cost; cost benefit analysis model to predict the SAF or jet fuel price; regression models to evaluate the cost for co-product such as diesel and petroleum in relation to SAF price. Estimated SAF, gasoline and diesel cost for the feedstock such as carinata oil, soybean oil, yellow grease and brown grease feedstock is included in the data.

## Specifications Table

**Table:** 

Subject	Economics
Specific subject area	Techno-economic analysis (TEA) of a sustainable aviation fuel production pathway.
Type of data	Text, Tables, Figures, Excel Spreadsheet
How data were acquired	Data was acquired from secondary data sources including (i) techno-economic analysis studies on renewable fuel productions (ii) publicly available report on cost for utilities such as electricity, water and natural gas (iii) US refiner petroleum product price (iv) public report on feedstock price such as vegetable oil (carinata and soybean oil); yellow grease and brown grease price (iv) Experimental study on CH process (v) Employment cost index for total compensation for private industry workers by occupational group and industry (vi) TEA Evaluation model form the previous studies (vii) Plant design and economics for chemical engineers
Data format	Raw Analysed
Parameters for data collection	The model considered is a TEA of CH in the cost year 2017. Data required were equipment cost for three different processing that includes preconditioning unit, conversion unit and hydrotreating and fractionation unit; price of vegetable oil such as carinata oil and soybean oil; price of waste greases such as yellow grease and brown grease; price for petroleum-based fuel such as gasoline, diesel, and jet fuel; cost for the chemicals/catalyst; CH fuel yields; Gasoline, diesel and jet fuel density; green field fuel processing plant ratio factor based on equipment cost; operating labour cost.
Description of data collection	Equipment cost were estimated using literature data from the process with similar process conditions [1,2]. Historic price data for petroleum-based fuels such as gasoline, diesel, and jet fuel [3]. Cost of an oil seed processing plant for estimating carinata oil cost was adopted from the camelina oil seed processing study [4]. Soybean oil and yellow grease price were from U.S. State Department of Agriculture [5]. Equipment process conditions, CH process flow, fuel yield and fuel cuts for economic analysis were used from the CH experimental study [6], [7], [8]. Gasoline, diesel and jet fuel density were adopted from the technical review report on biodiesel conversion technologies [9]. Green field fluid processing plant ratio factor for estimating the capital cost based on the delivered equipment cost from Plant design and economics for chemical engineers [2]. Chemical plant operating labour cost from [10].
Data source location	Primary data sources (resources for the secondary data used in this analysis): Patent and Experimental article for Catalytic Hydrothermolysis [6,7] US Average Annual Industrial Electricity and Natural gas rate [11,12] USDA oil crop and yellow grease cost [5] Review studies on the biofuel conversion pathways [9] Chemical Plant design and economics [2,10,13] Techno-economic analysis studies on renewable fuel productions [1,4,8,10,14] US Refiner Petroleum Product Prices by Sales, Sales for Resale [3] Employment Cost Index Historical Listing – Volume III National Compensation Survey, Table 5[15] Hydrogen Cost [16] Producer Price Index of Commodity Price: Chemicals and Allied Products [17] Chemical Engineering Magazine Plant Cost Index [18]
Data accessibility	with the article Instructions for accessing these data: Supplementary data in related research article: https://ars.els-cdn.com/content/image/1-s2.0-S1364032121007954-mmc1.zip
Related research article	Sudha Eswaran, Senthil Subramaniam PhD, Scott Geleynse PhD, Kristin Brandt, Michael Wolcott PhD, Xiao Zhang PhD, Techno-economic analysis of catalytic hydrothermolysis pathway for jet fuel production. Renewable and Sustainable Energy Reviews, 2021. 151: p. 111516, https://doi.org/10.1016/j.rser.2021.111516.

## Value of the Data

• The dataset provides detailed economic data for a chemical plant to perform economic assessment of CH SAF production pathway. The data includes equipment cost for individual processing units. Model evaluation is automated based on the feedstock chosen.
• This dataset may be used in future studies and academic review on techno-economic analysis of SAF pathways, e.g. to estimate the fuel price for the conversion of different oil feedstock to jet fuel, adopting cost for processing units, evaluating co-product price in relation to jet fuel price by using regression analysis.
• Cost benefit analysis is implemented in this TEA worksheet. The model worksheet can be reused to evaluate TEA with the change of delivered equipment cost and respective operating cost for any of SAF conversion pathway. Pilot scale and commercial scale production capacity can be conFig.d and calculate the minimum selling price of SAF for the scaled capacity.

## 1 Data Description

Secondary data from other sources and the primary data or the plant cost estimates used to build a TEA model of CH SAF pathway for the cost year 2017 is presented in this dataset. This dataset supports the original research on accessing the economic viability of the CH SAF pathway for commercial scale production of 832 metric ton per day.

Table 1 provides the assumed economic parameters for the n^th^ plant economic analysis.

**Table 1:** Assumed economic parameters for the TEA model.

Economic parameters	Assumed values
Cost Year	2017
Feedstock to mill gate (MT/day)	832
Plant financing	30% equity, 70% loan
Loan rate	8%
Loan term	10 years
Plant life	20 years + 3 years for construction
Income tax rate	17.2%
Inflation	2%
Working capital	20% annual operating costs
Depreciation schedule	7 years [19], double declining balance to straight line
Construction schedule	3 years (8%, 60% and 32% of FCI for years 1,2 and 3, respectively)
Real discount rate	10%
Nominal Discount Rate	12.2%
Operations days/year	329 (90% uptime) [10]

Table 2 provides the information on the Input parameters used for the TEA model. This includes the price of utilities such as Electricity, Natural gas, and water. Feedstock price per MT for Soybean oil, carinata oil, yellow grease, and brown grease. Table includes all the configurable data for the model.

**Table 2:** Input parameters.

Item	Value	Source
Cost year	2017	
k MT/yr to process	273	
MT/day Feedstock to mill gate	832	
Feedstock Loss (%)	0%	
Days per year	329	[10] (90% up-time)
Hours per day	24	
Electricity cost ($/kwh)	$0.069	[11]
Natural gas cost ($/k cf)	$4.3	[12]
Natural gas cost ($/MMBtu)	$4.18	[12]
Cooling Water Cost ($/kg)	$0.00002	[13]
Inflation Rate	2.0%	
Hydrogen Cost ($/MT)	$1,740	[16]
Hydrocarbon Yield (kg/kg Oil)	0.63	
Oil to CH Crude Yield (kg/kg)	0.85	[7]
CH Oil to HC Yield (kg/kg)	0.72	[7]
Jet fuel yield	0.3681	
Jet Fuel Density (kg/L)	0.80	[9]
Gasoline Density (kg/L)	0.77	[9]
Gasoline Cut	0.2525	[7]
Gasoline Price ($/liter)	$1.22	Regressed data
Diesel Density (kg/liter)	0.84	[9]
Diesel Cut	0.2794	[7]
Diesel Price ($/liter)	$1.34	Regressed data
Feed stock prices ($/metric ton)		
Carinata Oil	$701	
Soybean Oil	$791	[5]
Yellow grease	$473	[5]
Brown Grease	$595	Estimated from [5] and [20]
Plant scenario	200,000	Assumption
Model scale	200,000	Assumption

**Table 3:** Electricity consumption and Cost per year.

Unit	kW	kWh/Yr.	Cost ($/Yr.)	Source
Pre-conditioning & CH	2222	17519431	$1,203,497	[21]
Hydrotreating & Distillation	697.1	5496094	$377,554	[21]

**Table 4:** Cooling water consumption and Cost per year.

Unit	Rate (lb./min)	kg/yr.	Cost ($/Yr.)	Source
Pre-conditioning & CH	11597.22	2488382556	$41,941	[7]
Hydrotreating & Distillation	21876	4693804669	$79,112	[22]

**Table 5:** Natural gas consumption and Cost per year.

Unit	Rate (BTU/hr.)	MMBtu/yr.	Cost ($/Yr.)	Source
Hydrotreating and Distillation	162205002	1278824	$5,349,168	[1]

**Table 6:** Hydrogen and Catalyst cost per year.

Item	Rate (MT/day)	MT/yr.	$/MT	Cost ($/Yr.)	Source
Hydrogen	1.730185052	568	$1,740	$988,956	[7]
Hydrotreating Catalyst	0.646395412	212.3	$33,200	$7,047,626	[1,17]
Preconditioning Catalyst	0.075978995	25.0	$1,800	$44,926	[23,24]
CH Catalyst	0.025326332	8.3	$1,500	$12,480	[7,24]

**Table 7:** Fixed operating cost per year.

Fixed Operating Costs	Cost (MM$/year)	Source
Maintenance	$8.8	6% FCI
Labor + Benefits	$2.9	[10]
Taxes and Insurance	$3.7	2.5% FCI

**Table 8:** Preconditioning (Catalytic conjugation & cyclization) Equipment cost for Carinata oil feedstock.

Equipment	Quantity	Equipment Cost, 2017$	Scaled Equipment Cost, 2017$
Feed Pumps	2	$47,400	$94,800
Reactors	2	$375,400	$750,800
Heat Exchanger	2	$124,200	$248,400

**Table 9:** Distillation unit equipment cost.

Equipment	Purchased Cost, 2002$	Scaled Purchased Cost, 2017$	Source
Distillation unit	$800,000	$1,042,690	[2]

**Table 10:** Conversion (Catalytic Hydrothermolysis) Equipment cost. Grease cleanup cost is estimated for waste grease processing.

Equipment	Quantity	Scaling stream	Stream flow unit	Referred Equipment stream flow	New Flow	Size ratio	Referred equipment cost	Base Year	Scaling exponent	Scaled equipment cost in base year	Scaled equipment cost in 2017$	Source
Clean-up reactor	1	Volume	gal	350	278	0.79	$426,275	2014	0.56	$374,526	$368,935	[1]
Feed pump	2	Feed Flow rate	gal/min	69	139	2.01	$196,819	2014	0.33	$247,929	$488,456	
Heater	2	duty	mmBtu/hr	5.2	4.1	0.79	$275,289	2014	0.7	$234,169	$461,347	
Pressure regulator (valve)	3	Feed flow rate	gal/min	138.89	139	1.00	$61,600	2017	0.7	$61,600	$184,799	
Feed Mixer	1	Area	ft2	1284	1019.05	0.79	$3,071,695	2014	0.7	$2,612,880	$2,573,875	[1]
CH Reactor	1	Volume	gal	350	278	0.79	$426,275	2014	0.56	$374,526	$368,935

**Table 11:** Post-refining (Hydrotreating & Distillation).

Equipment	Scaling stream	Stream flow unit	Referred Equipment stream flow	New Flow	Size ratio	Referred equipment cost	Base Year	Scaling exponent	Scaled equipment cost in base year	Scaled equipment cost in 2017$	Source
Hydrotreater Reactor, vessels, columns	Feed volume	gal/min	79.7	139	1.74	$13,904,784	2014	0.75	$21,093,050	$18,878,303	[1]

**Table 12:** Capital Cost Estimation for Carinata oil feedstock.

Process Area		Delivered Equipment Cost, MM$	Total Capital Investment, MM$	Source
Pre-conditioning	ISBL	$1.2		
Catalytic Hydrothermolysis	ISBL	$4.5		
Hydrotreating & Distillation	ISBL	$21.9		
**Total Equipment Cost**		**$27.6**		
**Total Direct Costs (TDC)**			**$106.8**	**Ratio Factor = 3.87 [****2****]**
**Fixed Capital Investment (FCI)**			**$146.6**	**Ratio Factor = 5.31 [****2****]**
**Total Capital Investment (TCI)**			**$191.0**	**FCI + WC**

Operation cost estimated for the model is detailed in the Table 3, Table 4, Table 6 and 7, this includes cost estimation for the utilities, chemical and catalyst, fixed operation cost for the plant for one-year period.

Equipment cost estimation for the three processing units such as preconditioning, CH conversion and post refining step includes hydrotreating and distillation unit costs. Table 8, Table 9, Table 10 and 11 details the estimated equipment cost based on the model scale for carinata oil feedstock. Processing waste grease feedstock such as brown grease or yellow grease do not include preconditioning cost.

Capital investment was estimated on the greenfield fluid processing ration factor from Plant design and Economics for chemical engineer hand book [2]. Estimated capital cost is presented in the Table 12.

Regression over historic fuel price [3] to evaluate the cost of co-products such as gasoline and diesel in relation to jet fuel price.

**Fig. 1:**
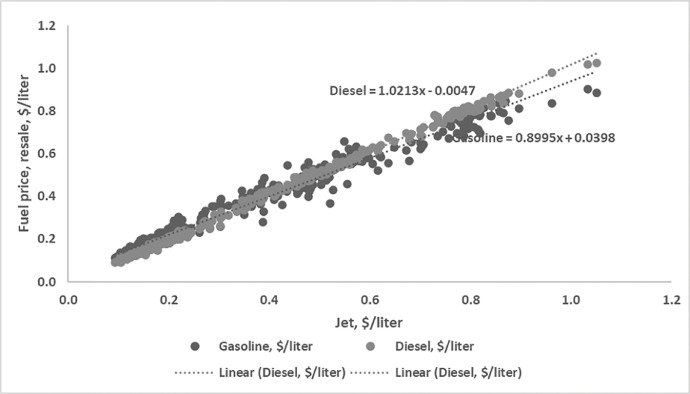
Regression over historic petroleum-based fuels

Annual production quantity and the estimated jet fuel price per litre and regressed fuel price for diesel and gasoline based on equation in Fig. 1 is shown in the Table 13 below.

**Table 13:** Annual production quantity (MML/ yr.) and fuel cost ($/L) for Carinata oil feedstock.

Product	Annual Product	Units	Price $/liter
Jet Fuel	79	MM liter/yr.	**$1.32**
Gasoline	56	MM liter/yr.	**$1.22**
Diesel	57	MM liter/yr.	**$1.34**

**Fig. 2:**
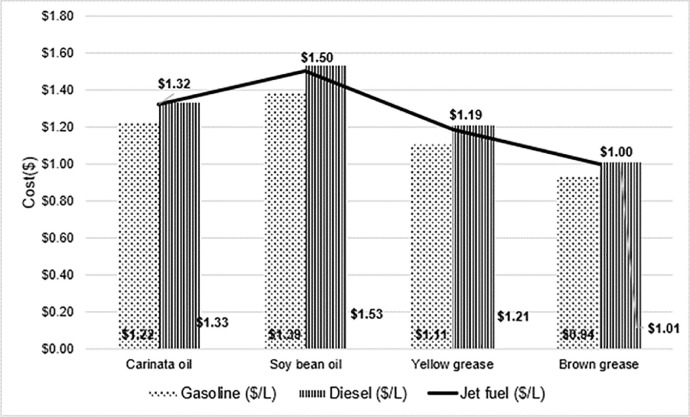
Estimated SAF, Gasoline, Diesel price($/L) for four different feed stock

Estimated gasoline, diesel cost in relation with SAF minimum selling price for four selected feedstock such as Carinata oil, Soybean oil, Yellow grease and Brown grease is shown in Fig. 2.

## 2 Experimental Design, Materials and Methods

The economic feasibility of a biofuel pathway depends on the combination of capital and raw material costs, availability of raw materials as well as other operational costs. Ratio factors were used to determine outside battery limits (OSBL) costs from inside battery limits (ISBL) equipment costs. ISBL equipment is integral to a specific process while OSBL equipment support the core process and include processes like steam generation, waste water treatment and buildings [2]. Equipment scale was estimated and used to scale the cost using the exponential correlation [1,2]. This cost was unified to 2017 dollars using the Chemical Engineering Plant Cost Index [18]. The ratio factor for a greenfield liquid processing plant was applied to the equipment costs, to estimate the direct costs and the fixed capital investment. The total capital investment (TCI) is the sum of the fixed capital investment (FCI) and the working capital. Working capital, which is used to cover operating costs when the facility is not able to cover expenses, is assumed to be 20% of the annual operating costs. Land cost is assumed to be 1.5% of the TCI [25].

In the analysis, the production plant for CH pathway is assumed to depreciate in 7 years, following double declining balance to straight line, and the plant life is 20 years. The project is assumed to be 30% equity financed and 70% loan with loan term for 10 years. For the present cost analysis, the fixed capital investment is spread over 3 years at a rate of 8%, 60% and 32% respectively. A cost benefit analysis was used to evaluate the economic feasibility of the CH process by predicting the minimum selling price (MSP) of SAF. MSP per unit volume of SAF is defined as the price that has a net present value (NPV) of zero and nominal financial discount rate of 12.2%. We assume an inflation rate of 2% following the average inflation from 1997 to 2017. The inclusion of inflation in the economic analysis, which combines the real discount rate of 10% with inflation to determine the nominal discount rate of 12.2%.

## CRediT authorship contribution statement

**Sudha Eswaran:** Methodology, Formal analysis, Investigation, Writing – review & editing. **Senthil Subramaniam:** Methodology, Formal analysis, Investigation. **Scott Geleynse:** Data curation, Validation. **Kristin Brandt:** Data curation, Validation, Writing – review & editing. **Michael Wolcott:** Conceptualization, Supervision. **Xiao Zhang:** Conceptualization, Data curation, Validation, Writing – review & editing, Supervision.

## Declaration of Competing Interest

The authors declare that they have no known competing financial interests or personal relationships which have or could be perceived to have influenced the work reported in this article.
